# Expression of vitamin D receptor, CYP24A1, and CYP27B1 in normal and inflamed canine pancreases

**DOI:** 10.3389/fvets.2023.1265203

**Published:** 2023-09-21

**Authors:** Dohee Lee, Sanggu Kim, Yoonhoi Koo, Yeon Chae, Juwon Wang, Soochong Kim, Taesik Yun, Mhan-Pyo Yang, Byeong-Teck Kang, Hakhyun Kim

**Affiliations:** ^1^Laboratory of Veterinary Internal Medicine, College of Veterinary Medicine, Chungbuk National University, Cheongju, Republic of Korea; ^2^Laboratory of Veterinary Pathology and Platelet Signaling, College of Veterinary Medicine, Chungbuk National University, Cheongju, Republic of Korea

**Keywords:** CYP24A1, CYP27B1, dog, immunohistochemistry, pancreatic tissue, pancreatitis, vitamin D receptor

## Abstract

Vitamin D plays a role in anti-inflammatory processes, and the alteration of its metabolism is associated with the inflammatory processes of pancreatitis. This study was performed to evaluate the expression of the vitamin D receptor (VDR) and the two major enzymes that regulate vitamin D metabolism, 1α-hydroxylase (CYP27B1) and 24-hydroxylase (CYP24A1), in the canine pancreas and to compare their degrees of immunoreactivity between normal and inflamed pancreases. Five normal and inflamed pancreatic tissues each were obtained from six dogs. The expression of VDR, CYP24A1, and CYP27B1 were determined immunohistochemically, and the degree of immunostaining was assessed semiquantitatively. The VDR was expressed in the ducts, acini, and islets of Langerhans of normal pancreases and in the ducts and acini of inflamed ones. There was a significant difference in the immunoreactivity score for VDR in the islets of Langerhans between normal (median, 3 [interquartile range, 2–7.5] score) and inflamed pancreatic tissues (0 [0–0.5] score, *p* = 0.03). CYP24A1 was expressed in the ducts and islets of Langerhans in both normal and inflamed pancreases, whereas CYP27B1 was expressed in the ducts and acini in only some normal and inflamed pancreatic tissues. This study showed that VDR expression decreased in inflamed pancreases and demonstrated CYP24A1 and CYP27B1 expression in the canine pancreas for the first time. These findings indicate that the pancreas could regulate the metabolism and biological activity of vitamin D and suggest that a decrease in these might be related to the pathophysiology of pancreatitis.

## 1. Introduction

Pancreatitis, an inflammatory disease of the pancreas, is the most common exocrine pancreatic disease in dogs (1). Clinical signs include vomiting, abdominal pain, and anorexia in dogs, and it could lead to systemic inflammatory response syndrome or multiple organ dysfunction syndromes, which are potentially life-threatening (1, 2). It is facilitated by innate or adaptive immune responses (3) and various etiologies and risk factors of pancreatitis have been suggested (4). Of which the alteration of vitamin D metabolism is suggested to be associated with inflammatory processes of pancreatitis in rodents and humans, presumably due to vitamin D’s biological role including anti-inflammation, immunoregulation, and anti-fibrotic effects (5–8). However, information on the association between canine pancreatitis and the alteration of vitamin D metabolism is limited.

Vitamin D metabolism is regulated through complex processes involving two major enzymes (6). In the kidney, 1α-hydroxylase (CYP27B1) converts 25-hydroxyvitamin D [25(OH)D] to 1,25-dihydroxyvitamin D [1,25(OH)_2_D], a biologically active form that performs its biological functions by binding to the vitamin D receptor (VDR) in target tissues (6, 9). The enzyme 24-hydroxylase (CYP24A1), one of the main target genes of the VDR (10, 11), is upregulated by activated VDR and converts vitamin D metabolites into a biologically inactive form (12). Therefore, the biological activity of vitamin D is determined by the degree of VDR, CYP24A1, and CYP27B1 expression (13).

Previous studies revealed that the VDR is expressed in the canine pancreas (14) and serum VDR concentrations decreased in dogs with acute pancreatitis (15). These findings suggest that pancreatitis could possibly be linked to changes in VDR expression in canine pancreatic tissues; however, no studies have investigated VDR expression in inflamed canine pancreases. In addition, the expression of CYP24A1 and CYP27B1, principally abundant in the kidneys, was reported in the pancreas of humans (16–19); however, their expression in the pancreatic tissue in dogs has not been investigated. Considering that VDR is present in the canine pancreas and CYP24A1 and CYP27B1 are closely involved in vitamin D metabolism and its biological functions, the expression of these enzymes in canine pancreatic tissues are worth investigating.

The objective of this study was (1) to evaluate the expression of VDR, CYP24A1, and CYP27B1 in normal and inflamed pancreatic tissue in dogs and (2) to compare the degree of immunoreactivity between normal and inflamed pancreases using a semiquantitative method. We hypothesized that inflamed pancreatic tissue would show less VDR expression than that in normal pancreatic tissue and that CYP24A1 and CYP27B1 would be expressed in canine pancreases.

## 2. Materials and methods

### 2.1. Sample procurement

This study was approved by the Ethics Committee of Chungbuk National University, South Korea (CBNUA-1988-22-01). Pancreatic tissue samples were collected from six dogs that underwent partial pancreatectomy, were euthanized, or died for various reasons at the Chungbuk National University Veterinary Teaching Hospital. Clients of each dog provided informed consent for donating and storing tissue samples for future research. In some dogs, blocks were collected from two pancreatic sites (pancreatic head and body/tail) if their appearance was grossly different; 10 pancreatic tissue samples from six dogs were collected (Table 1). All samples were obtained within 2 h of euthanasia or death to reduce the influence of autolysis and fixed in 10% formalin. The obtained samples were formalin-fixed and paraffin-embedded according to standard methods (20).

**Table 1:** Signalments of dogs and characteristics of samples.

Group	Number (dogs)	Breed	Sex	Age	Diagnosis	Sample acquisition	Number (samples)	Histopathological grade
Control	Dog 1^*^	Maltese	SF	5 y 10 m	Brain infarction	Necropsy (after euthanasia)	1	Normal
							2	Normal
	Dog 2^*^	Golden retriever	IM	4 y 11 m	Idiopathic epilepsy	Necropsy	3	Normal
							4	Normal
	Dog 3	Maltese	CM	6 y	MUE	Necropsy	5	Minimal inflammation
Pancreatitis	Dog 4	Miniature poodle	IF	14 y	Ependymal cyst	Necropsy	6	Mild pancreatitis
	Dog 5^*^	Jindo	CM	12 y 5 m	Pancreatitis	Partial pancreatectomy	7	Severe pancreatitis
							8	Severe pancreatitis
	Dog 6^*^	Miniature poodle	CM	8 y 7 m	Choroid plexus tumor	Necropsy	9	Severe pancreatitis
							10	Moderate pancreatitis

^*^Samples were collected from two pancreatic sites (pancreatic head and body/tail) because the appearance at them was grossly different. For dog ages; y, years; m, months; CM, castrated male; IF, intact female; IM, intact male; MUE, meningoencephalitis of unknown origin; SF, spayed female.

### 2.2. Histopathological evaluation

All formalin-fixed, paraffin-embedded tissue blocks were sectioned into 5 μm-thick slices and stained with hematoxylin (Harris Hematoxylin; BBC Biochemical, WA, United States) and eosin (Eosin Y; BBC Biochemical).

When histopathological changes were identified in pancreatic tissue, including neutrophilic and lymphocytic inflammation, pancreatic necrosis, peripancreatic fat necrosis, edema, fibrosis, and atrophy, the sample was classified as having pancreatitis (21). In all tissue samples classified as having pancreatitis, the type of infiltrated inflammatory cells was recorded, and the semiquantitative histopathological grade was assessed based on the surface area affected by a lesion. When <10%, 10–40, and > 40% of the evaluated section were affected by a lesion, the sample was classified as having mild, moderate, and severe pancreatitis, respectively (21).

When the histopathological changes were absent or minimal changes were observed, the samples were defined as being normal pancreases.

### 2.3. Immunohistochemistry

Antibodies against VDR (MA1-710; Thermo Fisher Scientific, CA, United States), CYP24A1 (PA5-21704; Thermo Fisher Scientific), and CYP27B1 (ab206655; Abcam, MA, United Kingdom) were used for immunohistochemistry (Table 2). Five micrometer-thick sections from each formalin-fixed, paraffin-embedded block were prepared on silane-coated slides. After deparaffinizing and rehydration with xylene and alcohols, slides were washed under tap water for 10 min. Antigen retrieval was performed by boiling washed slides in Tris-EDTA buffer (pH 9.0) for 10 min in a microwave and kept at room temperature (RT) for 30 min. Then, antigen-retrieved slides were washed and incubated with 3% H_2_O_2_ for 10 min at RT. The washed slides were blocked with 5% goat serum (Vector Laboratories, CA, United States) in phosphate-buffered saline (PBS) for 1 h, followed by washing with PBS. The slides were then incubated with primary antibodies (Table 2) at 4°C overnight. After washing the slides with PBS, they were incubated with diluted secondary antibodies for 30 min at RT secondary antibodies according to the manufacturer’s instructions. Anti-rat secondary antibody (Vectastain® ABC Kit, PK-6104; Vector Laboratories) was used for VDR and anti-rabbit secondary antibody (Vectastain® ABC Kit, PK-6101; Vector Laboratories) were used for CYP24A1 and CYP27B1. The dilution ratio for the secondary antibodies were 1:200. Slides were washed in PBS and incubated with ABC reagent (Vectastain® ABC Kits, PK-4000; Vector Laboratories) for 30 min at RT. After washing the slides again with PBS for 5 min, visualization was performed by incubating them in 3,3′-diaminobenzidine tetrahydrochloride solution (Vector® DAB Substrate Kit, SK-4100; Vector laboratories) for 10 min. The slides were washed and counterstained with hematoxylin for 1 min. The negative control tissue sections were stained with PBS instead of the primary antibody and normal kidney tissue paraffin sections were used as positive controls (Supplementary material).

**Table 2:** List of antibodies used for immunohistochemistry.

Antibody	Supplier	Catalogue #	Antibody type	Dilution
VDR	Thermo Fisher Scientific, CA, United States	MA1-710	Mouse monoclonal	1:100
CYP24A1	Thermo Fisher Scientific	PA5-21704	Rabbit polyclonal	1:500
CYP27B1	Abcam, Cambridge, MA, United Kingdom	ab206655	Rabbit monoclonal	1:200

CYP24A1, 24-hydroxylase; CYP27B1, 1α-hydroxylase; VDR, vitamin D receptor.

### 2.4. Quantification of immunoreactive cells

The degree of immunostaining was assessed using a semiquantitative assay according to the immunoreactive score (IRS) described by Remmele and Stegner (22–24) (Table 3). All histological and immunohistochemical stained sections were scanned with a slide scanner (Olympus VS200 Slide Scanner, Tokyo, Japan) and evaluated by expert observers (SGK and SCK) using ImageJ software (National Institutes of Health, Bethesda, MD, United States). Analysis and interpretation of the IRS were performed by DL and HK. The stain intensity was scored as follows: 0 = no, 1 = weak, 2 = moderate, and 3 = intense reactions. The percentage of cells positively stained was scored as follows: 0 = <5%, 1 = 5–25%, 2 = 25–50%, 3 = 51–75%, and 4 = >75% of cells stained. The IRS was calculated by multiplying the stain intensity and percentage of cells and ranked in a range from 0 to 12. The IRS for each slide was classified as follows: 0–1, negative; 2–3, mild; 4–8, moderate; and 9–12, strongly positive staining. Each tissue sample was scored for immunoreactivity in three parts, namely, the ducts, acini, and islets of Langerhans.

**Table 3:** Immunoreactive scoring system [modified from Kaemmerer et al. (21)].

Intensity of staining	Percentage of positive cells	IRS (0–12)
0 = no reaction	0 ≤5%	0–1 = negative
1 = weak reaction	1 = 5–25%	2–3 = mildly positive
2 = moderate reaction	2 = 26–50%	4–8 = moderately positive
3 = intense reaction	3 = 51–75%	9–12 = strongly positive
	4 ≥ 75%	

IRS = score of the intensity of staining × score of percentage of positive cells. IRS, immunoreactive score.

### 2.5. Statistical analyses

Statistical analyses were conducted using the commercial statistical software, Prism 9 (GraphPad Software Inc., La Jolla, CA). The Mann–Whitney U-test was used to compare the IRS of each region between normal and inflamed pancreatic tissues. Statistical significance was set at *p* < 0.05.

## 3. Results

### 3.1. Study group

Ten pancreatic tissues were collected from six dogs with a median age of 7.3 years (range, 4.9–14 years). Signalments of the dogs and sample characteristics used in this study are shown in Table 1. Five samples obtained from three dogs were diagnosed as having pancreatitis (mild, *n* = 1; moderate, *n* = 1; severe pancreatitis, *n* = 3), and another five samples collected from three dogs were normal. In the control group, one dog with meningoencephalitis of unknown origin had been administered a combination of prednisolone, mycophenolate mofetil, and phenobarbital, another dog with idiopathic epilepsy had been administered phenobarbital, and the other had no history of medication before presentation. In the pancreatitis group, one dog with an ependymal cyst had been administered a combination of prednisolone, phenobarbital, and potassium bromide, another dog with choroid plexus tumor had been administered cyclophosphamide, prednisolone, and potassium bromide, and the other with pancreatitis had no drug history. In the pancreatitis group, two dogs had increased serum C-Reactive Protein (79.47 mg/dL and 170.98 mg/dL, respectively; VetChroma; Boditech Med Inc., Republic of Korea) and cPL (>1,000 μg/L in both; Vcheck cPL; BioNote, Republic of Korea) concentrations. Data was unavailable for the other dog.

### 3.2. Vitamin D receptor expression

**Figure 1:**
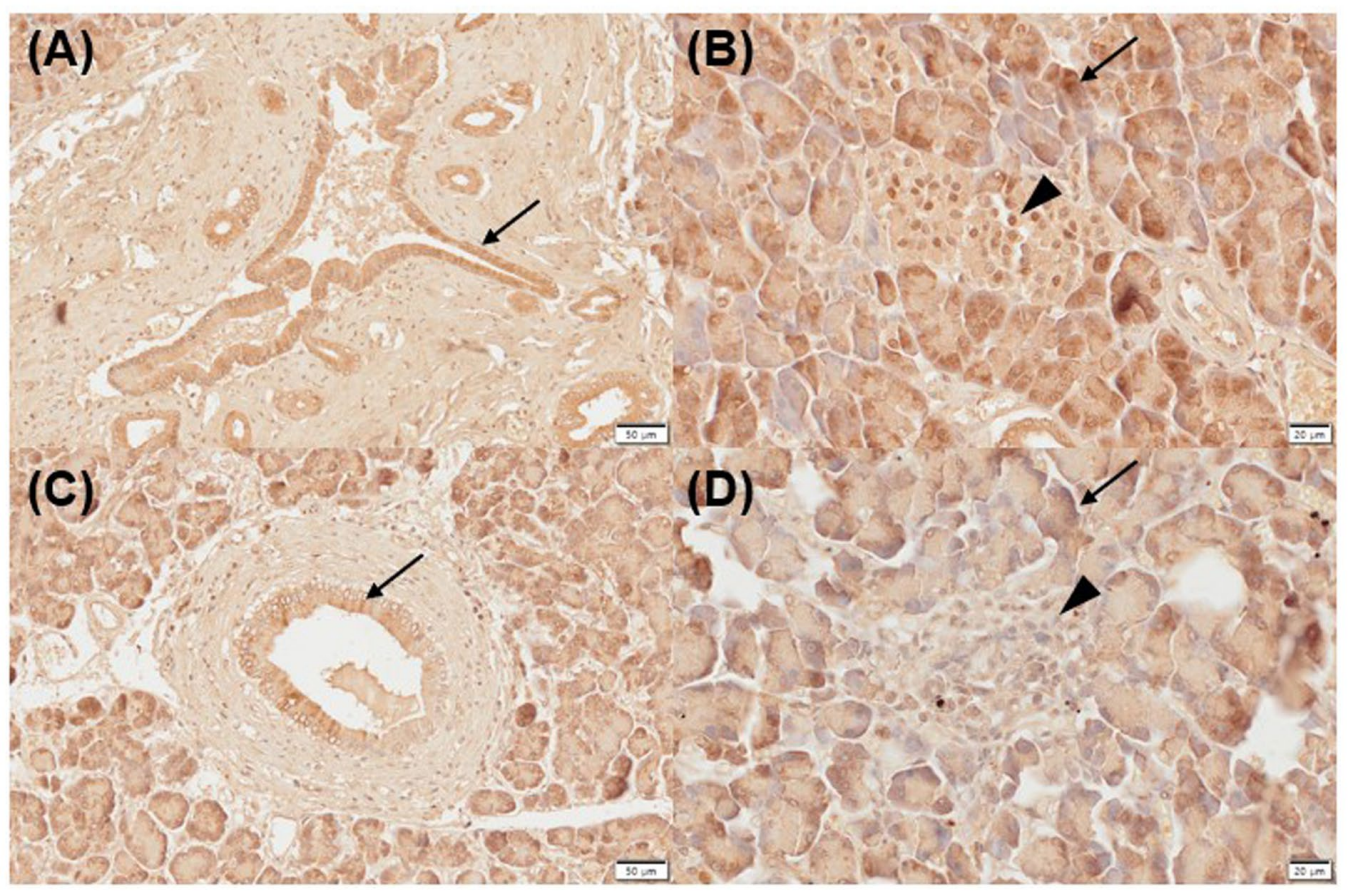
Immunohistochemistry expression of VDR in normal **(A,B)** and inflamed pancreases **(C,D)** in dogs. Normal pancreases were positive for VDR expression in pancreatic ducts [**(A)** arrow], acini [**(B)** arrow], and islets of Langerhans [**(B)** arrowhead]. Inflamed pancreases were positive for VDR expression in pancreatic ducts [**(C)** arrow] and acini [**(D)** arrow] but negative in islets of Langerhans [**(D)** arrowhead]. Scale bars: 50 μm **(A,C)** and 20 μm **(B,D)**. VDR, vitamin D receptor.

Three of five normal (moderate, *n* = 2; strongly positive, *n* = 1) and inflamed pancreas samples (mild, *n* = 1; strongly positive, *n* = 2) each showed positive immunoreactivity for VDR in the pancreatic ducts (Figures 1A,C). Two of five normal and inflamed pancreases each were strongly positive for VDR in the acini (Figures 1B,D). In the normal pancreas, 4/5 sections showed positive immunoreactivity (moderate, *n* = 3; strongly positive, *n* = 1) for VDR in the islets of Langerhans, and only one section was negative. However, all slides of inflamed pancreases showed negative immunoreactivity for VDR in the islets of Langerhans.

### 3.3. CYP24A1 expression

**Figure 2:**
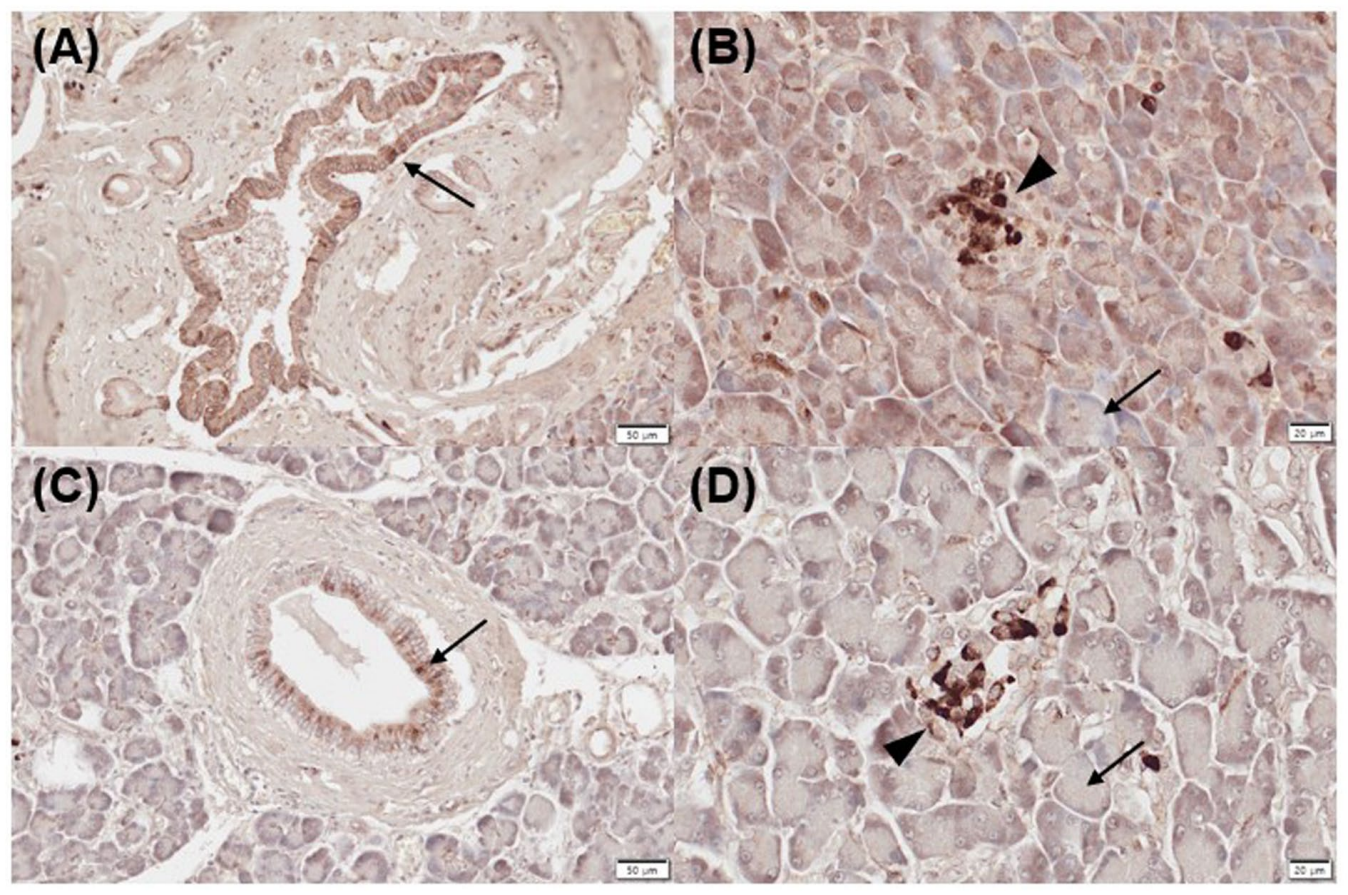
Immunohistochemistry expression of CYP24A1 in normal **(A,B)** and inflamed pancreases **(C,D)** in dogs. **(A)** Normal pancreases showed positive immunostaining for CYP24A1 in pancreatic ducts [**(A)** arrow] and islets of Langerhans [**(B)** arrowhead] but not in acini [**(B)** arrow]. Inflamed pancreases were also positive for CYP24A1 expression in pancreatic ducts [**(C)** arrow] and islets of Langerhans [**(D)** arrowhead] but not in acini [**(D)** arrow]. Scale bars: 50 μm **(A,C)** and 20 μm **(B,D)**. CYP24A1, 24-hydroxylase.

All normal (mild, *n* = 1; moderate, *n* = 3; strongly positive, *n* = 1) and inflamed pancreas samples (moderate, *n* = 3; strongly positive, *n* = 2) showed positive immunoreactivity for CYP24A1 in pancreatic ducts (Figures 2A,C), as well as negative immunolabelling for CYP24A1 in the acini (Figures 2B,D). All normal (moderate, *n* = 3; strong positive, *n* = 2) and 4/5 inflamed pancreatic samples (mild, *n* = 1; moderate, *n* = 2; strongly positive, *n* = 1) showed positive labelling for CYP24A1 in the islets of Langerhans (Figures 2B,D).

### 3.4. CYP27B1 expression

**Figure 3:**
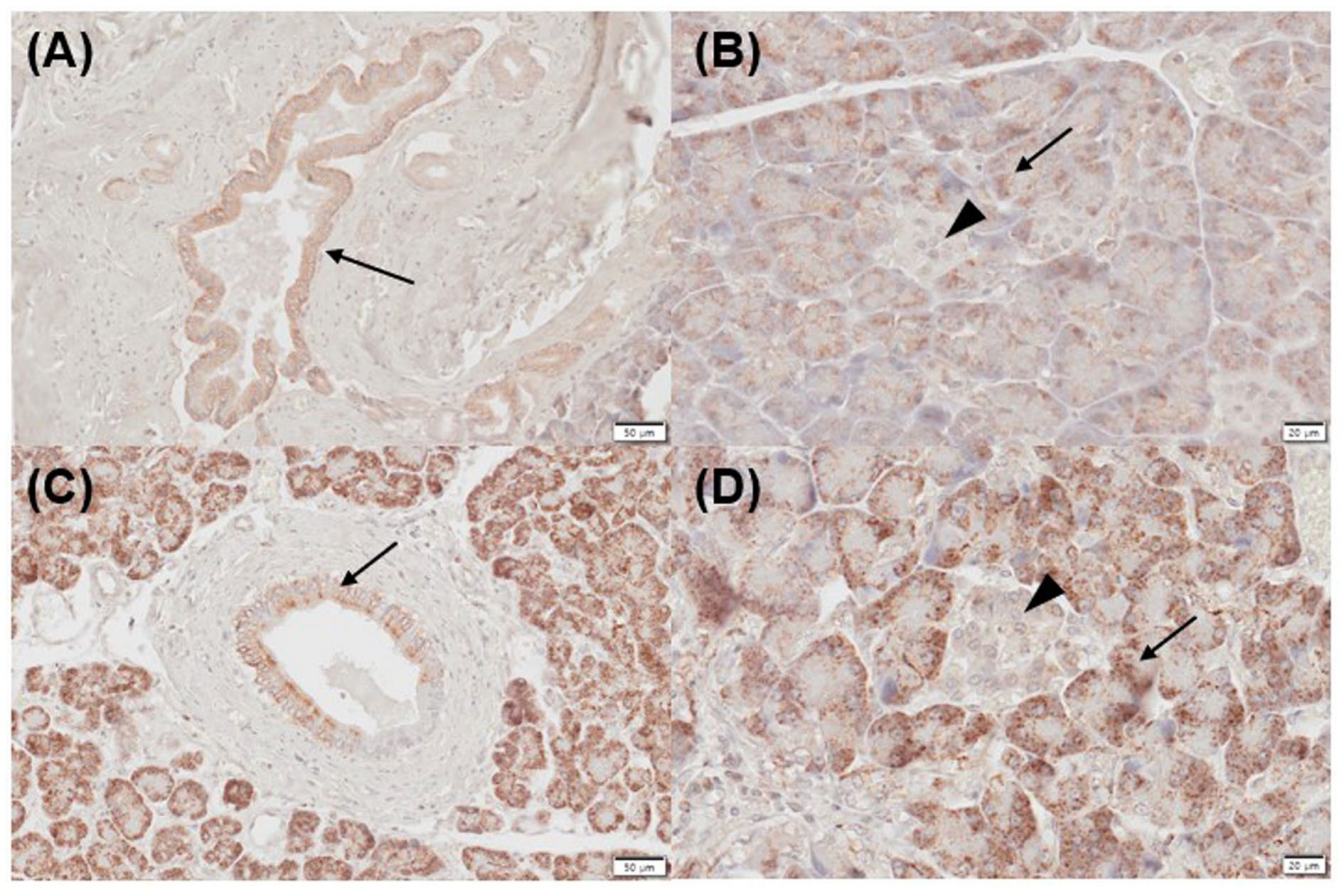
Immunohistochemistry expression of CYP27B1 in normal **(A,B)** and inflamed pancreases **(C,D)** in dogs. **(A)** Normal pancreases showed positive immunostaining for CYP27B1 in pancreatic ducts [**(A)** arrow] and acini [**(B)** arrow]. **(B)** CYP27B1 was immunolabelled in pancreatic ducts [**(A)** arrow] and acini [**(B)** arrow]. Most of the normal and inflamed pancreas samples showed no expression for CYP27B1 in the islets of Langerhans [**(B,D)** arrowhead]. Scale bars: 50 μm **(A,C)** and 20 μm **(B,D)**. CYP27B1, 1-α hydroxylase.

Three of five normal (mild positive, *n* = 3) and 2/5 inflamed pancreatic samples (mild, *n* = 1; moderate positive, *n* = 1) exhibited positive immunoreactivity for CYP27B1 in pancreatic ducts (Figures 3A,C). Three of five normal (mild, *n* = 1; moderate positive, *n* = 1; strong positive, *n* = 1) and 2/5 inflamed pancreases (strong positive, *n* = 2) showed positive immunolabelling for CYP27B1 in acini (Figures 3B,D). The islets of Langerhans did not show CYP27B1 expression in most normal and inflamed pancreatic tissues (Figures 3B,D), except for only 1/5 normal and inflamed pancreas each, which showed mild immunoreactivity.

### 3.5. Comparison of the immunoreactive scores for the vitamin D receptor, CYP24A1, and CYP27B1 in normal and inflamed pancreases

The IRS for VDR, CYP24A1, and CYP27B1 in each region is shown in Table 4 and Supplementary material. The median IRS for VDR in the islets of Langerhans was significantly different between the normal (median [interquartile range], 6 [2–7.5]) and inflamed pancreases (0 [0–0.5], *p* = 0.03). The median IRS for VDR in the pancreatic duct and acini was not significantly different between the normal and inflamed pancreases (*p* = 0.38 and *p* = 1.00, respectively). There was no significant difference in the median IRS for CYP24A1 and CYP27B1 in the duct (*p* = 0.21 and *p* = 0.65, respectively), acini (*p* = 0.55 and *p* = 0.91, respectively), and islets of Langerhans (*p* = 0.21 and *p* = 0.52, respectively) between the normal and inflamed pancreases.

**Table 4:** Immunoreactivity score for VDR, CYP24A1, and CYP27B1 in normal pancreas and inflamed pancreas.

		Normal pancreas (*n* = 5)	Inflamed pancreas (*n* = 5)	*p*-value
VDR	Duct	6 (0–7.5)	9 (1.5–9)	0.38
	Acini	0 (0–9)	0 (0–9)	1.00
	Islets of Langerhans	6 (2–7.5)	0 (0–0.5)	0.03^*^
CYP24A1	Duct	6 (3.5–7.5)	6 (6–9)	0.21
	Acini	0 (0–1)	1 (0–1)	0.55
	Islets of Langerhans	6 (6–9)	6 (1.5–7.5)	0.21
CYP27B1	Duct	2 (0–3)	0 (0–4)	0.65
	Acini	3 (0–7.5)	0 (0–9)	0.91
	Islets of Langerhans	0 (0–2)	0 (0–1)	0.52

Data are expressed as medians (interquartile range). *p < 0.05. CYP24A1, 24-hydroxylase; CYP27B1, 1-α hydroxylase; VDR, vitamin D receptor.

## 4. Discussion

The present study showed that the VDR is expressed in various regions of the pancreas in dogs, including the duct, acini, and islets of Langerhans, and its immunoreactivity significantly decreased in the islets of Langerhans of the inflamed pancreas. Furthermore, this study showed for the first time that CYP24A1 and CYP27B1 are expressed in the canine pancreas. Therefore, these findings suggest that the pancreas is involved in vitamin D metabolism in dogs by regulating related enzymes and that vitamin D activity would likely decrease in pancreatitis because of disrupted VDR expression.

VDR is widely distributed in the skeletal system and non-skeletal tissues, including the kidney, duodenum, ileum, and skin (25–27). VDR expression has also been documented in islets of Langerhans and acinar cells of human and rat pancreases (28); however, there is only one report of VDR expression in the canine pancreas (14). A previous study reported VDR expression in the endocrine and exocrine pancreas in dogs (14) and similarly, the present study showed that VDR is present in the islets of Langerhans, acini, and ducts of canine pancreases. The presence of VDR in the canine pancreas suggests a previously unknown role of the pancreas in vitamin D biological activity regulation and might systemically regulate calcium homeostasis and immune modulation.

A previous study showed that the serum VDR concentration is decreased in dogs with acute pancreatitis; however, it was unclear which organs contributed to the low serum VDR concentration (15). Although the present study included only pancreatic tissues and not others where VDR is highly expressed, such as the kidneys and gastrointestinal tract (25–27), it was shown that VDR expression was decreased in the inflamed pancreatic tissue, supporting that this could contribute to the low serum VDR concentrations in dogs with pancreatitis. Interestingly, VDR expression was disrupted in the islets of Langerhans, and no difference in immunoreactivity for VDR was found in the acini. This finding is unexpected, given that pancreatitis is an exocrine pancreatic disease initiated by the injury of acinar cells (6). Therefore, it would be reasonable to assume that VDR disruption in the endocrine pancreas could have been due to transcriptional regulation rather than cellular damage. VDR expression is regulated transcriptionally by various molecules, including 1,25(OH)_2_D, parathyroid hormone, and fibroblast growth factor 23 (29, 30), and differentially regulated in each tissue; for example, treatment with 1,25(OH)_2_D enhanced the VDR expression in kidneys but not in the intestine (30). Although the circulating levels of 1,25(OH)_2_D in dogs with pancreatitis were not determined, a decreased 25(OH)D concentration was reported in dogs with pancreatitis (15, 31) and might have negatively regulated *VDR* transcription in inflamed pancreases. It is unclear whether the VDR transcription in the island of Langerhans is more affected than that in the pancreatic ducts and acini by various factors such as 1,25(OH)_2_D. Further studies on the effect of circulating 1,25(OH)_2_D levels on VDR expression in each part of the pancreas (ducts, acini, and islets of Langerhans) would be beneficial to identify the pathophysiology of VDR disruption in inflamed canine pancreases.

VDR has anti-inflammatory effects, and the VDR/NLRP3 (nucleotide-binding oligomerization domain-like receptor family pyrin domain containing 3) pathway has been suggested to be linked to pancreatic inflammation (32, 33). When injured pancreatic acinar cells release intracellular contents, NLRP3 activates caspase-1 and stimulates inflammation (33). Activation of VDR with 1,25(OH)_2_D inhibits the NLRP3 inflammasome and reduces inflammatory cytokine expression (32). It is unknown whether the VDR present in endocrine islets could affect inflammatory cells to cause pancreatitis because research on its effect on inflammation has usually been limited to *in vitro* studies. However, considering a previous study of dogs with acute pancreatitis showing that a lower serum VDR concentration is associated with a higher serum inflammatory biomarker (15), it can be proposed that the disrupted VDR expression in inflamed pancreatic tissue could contribute to aggravating inflammation.

There is no specific treatment for pancreatitis. Therefore, treatments are generally confined to supportive care including fluid therapy for resuscitation or rehydration, anti-emetics, analgesics, and nutritional support (2, 34). The present study demonstrated that VDR expression is decreased in canine pancreatitis and suggests that VDR could be a potential target to treat pancreatitis in dogs. Previous studies revealed that supplementation of vitamin D increases *VDR* mRNA expression and VDR protein levels (35–37). Moreover, vitamin D could be used to prevent adverse effects caused by VDR reduction in canine pancreatitis. However, research on VDR expression at the protein level, in response to vitamin D administration, is limited to specific cells in certain disorders, such as fibroblasts in human patients with Crohn’s disease (36) and cerebral cortical cells in oxidative stress (37). To explore the potential of VDR as a target to treat pancreatitis in dogs, further studies assessing VDR expression and inflammation in the pancreas following vitamin D supplementation would be beneficial.

With the immunohistochemical analysis, the present study showed for the first time that CYP24A1 and CYP27B1 were present in the canine pancreas. *CYP24A1*, a target gene of VDR, encodes a degrading enzyme that converts 1,25(OH)_2_D into an inactive form and leads to vitamin D inactivation (11). In a human study, CYP24A1 of normal pancreases was principally localized in endocrine islets but not expressed in exocrine glands (18). Another human study showed that CYP24A1 of chronic pancreatitis tissues was primarily expressed in the endocrine islets, ducts, and acini (38). There has been no previous report comparing CYP24A1 expression in normal and inflamed pancreases in human and veterinary medicine. In this study, CYP24A1 was expressed in pancreatic ducts and the islets of Langerhans in the canine pancreas. Therefore, this finding suggests that the pancreas is involved in vitamin D metabolism in dogs. Furthermore, there was no difference in expressed regions or immunoreactivity of CYP24A1 between the normal and inflamed pancreases, and it seemed that inactivation of 1,25(OH)_2_D via CYP24A1 is not involved in the pathophysiology of pancreatitis. However, an increase in CYP24A1 expression in pancreatic tissue is associated with the development of pancreatic ductal adenocarcinoma in humans. This type of adenocarcinoma is explained by reduced 1,25(OH)_2_D activity that regulates tumoral processes such as proliferation, apoptosis, and angiogenesis (19, 37). Future research on CYP24A1 expression in canine pancreatic tumor might be beneficial to understand the role of vitamin D metabolism in the pathogenesis of canine pancreatic tumors, a pancreatic disorder that is uncommon but crucial in dogs.

CYP27B1 is classically presented in the kidney and located in various extra-renal tissues, including the skin, gastrointestinal tract, and pancreas (16, 17), and is involved in synthesizing the biologically active form of vitamin D. In the human pancreas, CYP27B1 is present in endocrine islets and ducts (16–18). This study showed that CYP27B1 was uncommonly expressed in endocrine islets of canine pancreases and was present in exocrine islets and pancreatic ducts of some pancreatic tissues. Considering that CYP27B1 is a vitamin D-activating enzyme, the exocrine pancreas would be closely involved in vitamin D activity not only by secreting digestive enzyme that promotes fat-soluble vitamin absorption (39) but also by converting 25(OH)D to its active form. In addition, it seemed that the conversion of the inactive form of vitamin D to its active form via CYP27B1 is not associated with the development of pancreatitis, considering that there was no difference in immunoreactivity of CYP27B1 between the normal and inflamed pancreases.

There were some limitations to the present study. Firstly, the sample size of each group was too small and therefore insufficient to elucidate the association between the severity of pancreatitis and the level of VDR expression. Two samples with mild to moderate pancreatitis had median IRS for VDR; 9 (interquartile range, 9–9), 4.5 (0–9), and 0.5 (0–1) in the pancreatic ducts, acini, and islets of Langerhans, respectively. In addition, three samples with severe pancreatitis had IRS of 3 (0–9), 0 (0–9), and 0 (0–0), respectively (data not shown). Although the median IRS for VDR tends to be lower in severe pancreatitis than in mild to moderate pancreatitis, there was no significant difference between the two groups (*p* = 0.2, 0.74, and 0.22 in ducts, acini and islets of Langerhans, respectively). However, the false negative results caused by type II error might have been found and results should be interpreted cautiously given the limited statistical power. Further study involving a large number of pancreatic tissue samples would be beneficial. Secondly, each dog had a different history of drug administration. In the six dogs included in this study, four were administered antiepileptic drugs and three with prednisolone. The phenobarbital-treated human hepatoma cell line showed upregulation of *CYP24A1* genes (40), and the dexamethasone-treated osteosarcoma cell line exhibited inhibition of *VDR* gene transcription (41). There is limited information on the effect of these drugs on VDR, CYP24A1, and CYP27B1 expression in the pancreas or *in vivo*, and a controlled study of drug history would be needed. Finally, this study did not compare acute and chronic pancreatitis, which might affect VDR, CYP24A1, and CYP27B1 expression levels.

In conclusion, this study showed that the VDR expression decreased in the islets of Langerhans during pancreatic inflammation and demonstrated the expression of CYP24A1 and CYP27B1 in canine pancreases for the first time. These findings suggest that the pancreas might regulate the metabolism and biological activity of vitamin D. In addition, our study establishes the potential of VDR as a target for canine pancreatitis treatment. Further studies that investigate the effect of vitamin D supplementation on VDR are needed to clarify whether vitamin D metabolism modification can ameliorate pancreatitis in dogs.

## Data availability statement

The original contributions presented in the study are included in the article/Supplementary material, further inquiries can be directed to the corresponding author.

## Ethics statement

This study was approved by the Ethics Committee of Chungbuk National University, South Korea (CBNUA-1988-22-01). Normal pancreatic tissues were obtained from a dog euthanized for a reason unrelated to the present study, which was approved by the Ethics Committee.

## Author contributions

DL: Conceptualization, Data curation, Formal analysis, Investigation, Methodology, Software, Writing – original draft. SaK: Investigation, Methodology, Software, Writing – original draft, Visualization. YK: Investigation, Data curation, Writing – review & editing. YC: Data curation, Investigation, Writing – review & editing. JW: Data curation, Investigation, Writing – review & editing. SoK: Investigation, Methodology, Software, Visualization, Writing – review & editing. TY: Writing – review & editing, Conceptualization, Formal analysis, Supervision. M-PY: Formal analysis, Supervision, Writing – review & editing, Validation. B-TK: Formal analysis, Supervision, Validation, Writing – review & editing, Conceptualization. HK: Conceptualization, Supervision, Writing – review & editing, Funding acquisition, Project administration.

## Funding

The author(s) declare financial support was received for the research, authorship, and/or publication of this article. This work was supported by the National Research Foundation of Korea (NRF) grant funded by the Korean Government (MSIT; No. NRF-2021R1F1A1061799).

## Conflict of interest

The authors declare that the research was conducted in the absence of any commercial or financial relationships that could be construed as a potential conflict of interest.

## Publisher’s note

All claims expressed in this article are solely those of the authors and do not necessarily represent those of their affiliated organizations, or those of the publisher, the editors and the reviewers. Any product that may be evaluated in this article, or claim that may be made by its manufacturer, is not guaranteed or endorsed by the publisher.

## Supplementary material

The Supplementary material for this article can be found online at: https://www.frontiersin.org/articles/10.3389/fvets.2023.1265203/full#supplementary-material

## Abbreviations

1,25(OH)2D: 1,25-Dihydroxyvitamin D
25(OH)D: 25-Hydroxyvitamin D
CYP24A1: 24-Hydroxylase
CYP27B1: 1α-Hydroxylase
IRS: Immunoreactive score
NLRP3: Nucleotide-binding oligomerization domain-like receptor family pyrin domain containing 3
PBS: Phosphate-buffered saline
RT: Room temperature
VDR: Vitamin D receptor
