# Supplementary material for: Expression of vitamin D receptor, CYP24A1, and CYP27B1 in normal and inflamed canine pancreases
Source: Front Vet Sci. 2023 Sep 21;10:1265203. doi: 10.3389/fvets.2023.1265203 (PMC10551448; doi:10.3389/fvets.2023.1265203)
Supplement: Supplementary file 1 [file Data_Sheet_1.DOCX]

Supplementary Material

Expression of Vitamin D Receptor, CYP24A1, and CYP27B1 in Normal and Inflamed Canine Pancreases

Dohee Lee^1^, Sanggu Kim^2^, Yoonhoi Koo^1^, Yeon Chae^1^, Juwon Wang^1^, Soochong Kim^2^, Taesik Yun^1^, Mhan-Pyo Yang^1^, Byeong-Teck Kang^1^, Hakhyun Kim^1*^

^1^Laboratory of Veterinary Internal Medicine, College of Veterinary Medicine, Chungbuk National University, Cheongju 28644, South Korea

^2^Laboratory of Veterinary Pathology and Platelet Signaling, College of Veterinary Medicine, Chungbuk National University, Cheongju 28644, South Korea

*** Correspondence:**Hakhyun Kim
[kimh@chungbuk.ac.kr](mailto:kimh@chungbuk.ac.kr)

# Supplementary Table

|  |  |  |  | VDR | | | | | | | | | CYP24A1 | | | | | | | | | CYP27B1 | | | | | | | | |
| --- | --- | --- | --- | --- | --- | --- | --- | --- | --- | --- | --- | --- | --- | --- | --- | --- | --- | --- | --- | --- | --- | --- | --- | --- | --- | --- | --- | --- | --- | --- |
| Group | Number (dogs) | Number (samples) | Histopathological grade | Duct | | | Acini | | | Islets of Langerhans | | | Duct | | | Acini | | | Islets of Langerhans | | | Duct | | | Acini | | | Islets of Langerhans | | |
|  |  |  |  | I | P | IRS | I | P | IRS | I | P | IRS | I | P | IRS | I | P | IRS | I | P | IRS | I | P | IRS | I | P | IRS | I | P | IRS |
| Control | Dog 1 | 1 | Normal | 0 | 0 | 0 | 0 | 0 | 0 | 2 | 3 | 6 | 2 | 3 | 6 | 1 | 1 | 1 | 3 | 2 | 6 | 0 | 0 | 0 | 0 | 0 | 0 | 1 | 1 | 1 |
|  |  | 2 | Normal | 2 | 3 | 6 | 0 | 0 | 0 | 2 | 2 | 4 | 2 | 3 | 6 | 1 | 1 | 1 | 3 | 3 | 9 | 0 | 0 | 0 | 0 | 0 | 0 | 0 | 0 | 0 |
|  | Dog 2 | 3 | Normal | 2 | 3 | 6 | 3 | 3 | 9 | 2 | 3 | 6 | 3 | 1 | 3 | 0 | 0 | 0 | 3 | 2 | 6 | 1 | 3 | 3 | 3 | 3 | 9 | 0 | 0 | 0 |
|  |  | 4 | Normal | 3 | 3 | 9 | 3 | 3 | 9 | 3 | 3 | 9 | 2 | 2 | 4 | 0 | 0 | 0 | 3 | 2 | 6 | 1 | 3 | 3 | 2 | 3 | 6 | 0 | 0 | 0 |
|  | Dog 3 | 5 | Minimal inflammation | 0 | 0 | 0 | 0 | 0 | 0 | 0 | 0 | 0 | 3 | 3 | 9 | 0 | 0 | 0 | 3 | 3 | 9 | 1 | 2 | 2 | 1 | 3 | 3 | 1 | 3 | 3 |
| Pancreatitis | Dog 4 | 6 | Mild pancreatitis | 3 | 3 | 9 | 0 | 0 | 0 | 0 | 0 | 0 | 2 | 3 | 6 | 1 | 1 | 1 | 0 | 0 | 0 | 0 | 0 | 0 | 0 | 0 | 0 | 0 | 0 | 0 |
|  | Dog 5 | 7 | Severe pancreatitis | 1 | 3 | 3 | 0 | 0 | 0 | 0 | 0 | 0 | 2 | 3 | 6 | 1 | 1 | 1 | 3 | 1 | 3 | 0 | 0 | 0 | 0 | 0 | 0 | 1 | 2 | 2 |
|  |  | 8 | Severe pancreatitis | 0 | 0 | 0 | 0 | 0 | 0 | 0 | 0 | 0 | 2 | 3 | 6 | 1 | 1 | 1 | 3 | 2 | 6 | 0 | 0 | 0 | 0 | 0 | 0 | 0 | 0 | 0 |
|  | Dog 6 | 9 | Severe pancreatitis | 3 | 3 | 9 | 3 | 3 | 9 | 0 | 0 | 0 | 3 | 3 | 9 | 0 | 0 | 0 | 3 | 3 | 9 | 2 | 1 | 2 | 3 | 3 | 9 | 0 | 0 | 0 |
|  |  | 10 | Moderate pancreatitis | 3 | 3 | 9 | 3 | 3 | 9 | 1 | 1 | 1 | 3 | 3 | 9 | 0 | 0 | 0 | 3 | 2 | 6 | 2 | 3 | 6 | 3 | 3 | 9 | 0 | 0 | 0 |

CYP24A1, 24-hydroxylase; CYP27B1, 1α-hydroxylase; I, intensity of staining; IRS, immunoreactive score; P, percentage of positive cells; VDR, vitamin D receptor

# Supplementary Figures

**
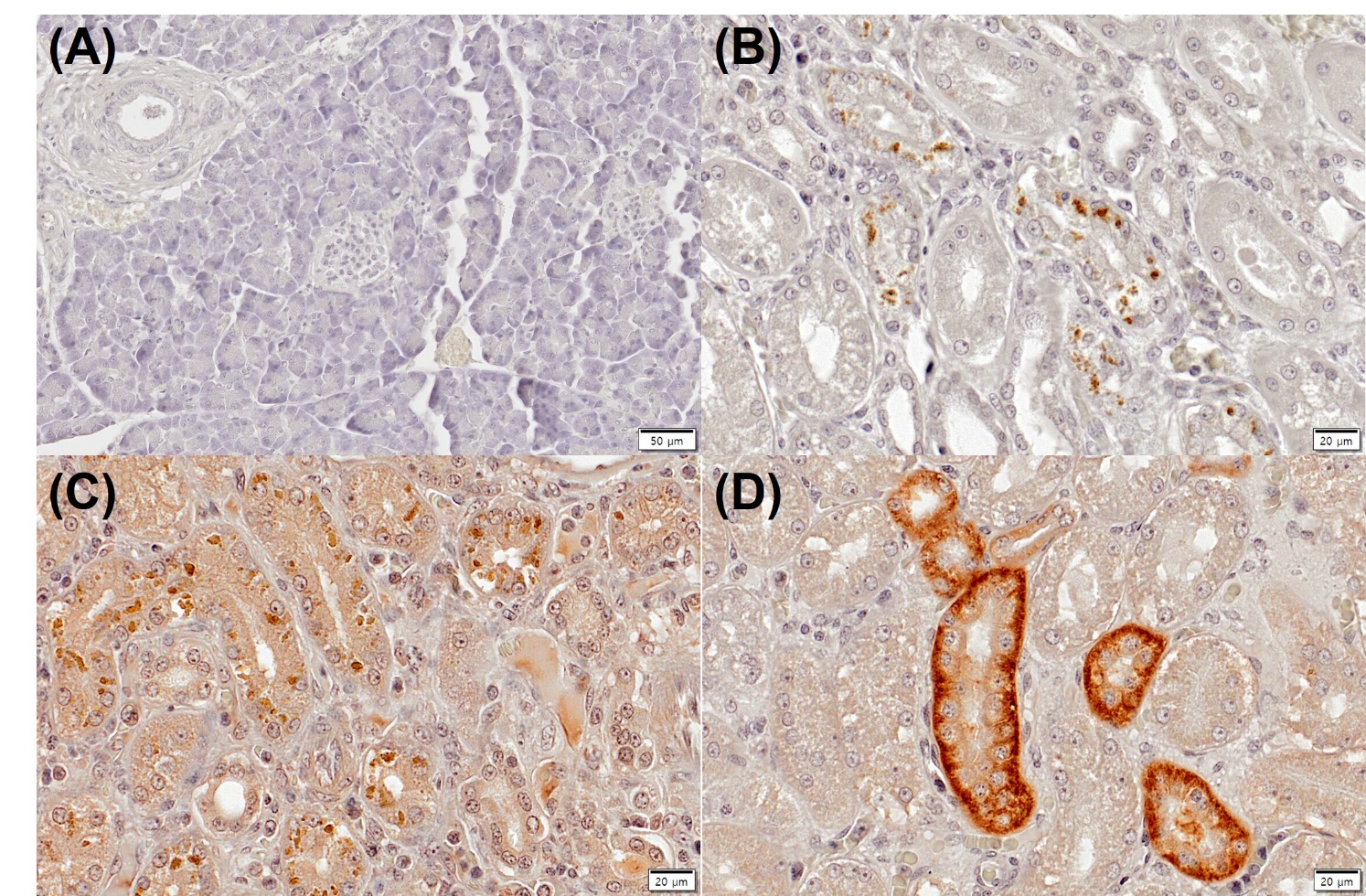
**

**Supplementary Figure 1.** For negative controls, tissue sections were stained with PBS instead of the primary antibody (A). Kidney tissues serve as positive controls for VDR (B), CYP24A1 (C), and CYP27B1 (D). Scale bars: 50 μm (A) and 20 μm (B, C, D). CYP24A1, 24-hydroxylase; CYP27B1, 1-α hydroxylase; PBS, phosphate-buffered saline; VDR, vitamin D receptor.
